# Mental Health Nurses' Attitudes and Perceptions Towards the Trauma Specialist Nurse Role: A Cross‐Sectional Study

**DOI:** 10.1111/jpm.70155

**Published:** 2026-06-02

**Authors:** Merav Ben Natan, Ira Sharon, Maya Carmon Klanss, Galit Levi

**Affiliations:** ^1^ Department of Nursing Tel Aviv University Tel Aviv Israel; ^2^ Hillel Yaffe Medical Centre, Academic School of Nursing Hadera Israel; ^3^ Lev Hasharon Mental Health Center Netanya Israel

**Keywords:** attitudes, mental health nurses, mental health nursing, professional role, trauma‐informed care

## Abstract

**Background:**

Trauma‐informed care (TIC) is widely recognised in psychiatric practice, yet the trauma specialist nurse role remains undefined in Israel.

**Aims:**

This study aimed to examine mental health nurses' attitudes and perceptions of the trauma specialist nurse role and to identify predictors of support for its implementation.

**Design:**

This study utilised a cross‐sectional research design.

**Method:**

This cross‐sectional study included 152 mental health nurses from inpatient, forensic, rehabilitation, and community settings. Data were collected using an adapted questionnaire based on the Attitudes Related to TIC Scale.

**Findings:**

Professional authority and responsibility (*β* = 0.643, *p* < 0.001) and TIC attitudes (*β* = 0.423, *p* < 0.001) were the strongest predictors of mental health nurses' perception of the trauma specialist nurse role, while trauma‐related knowledge and skills showed only a marginal association (*β* = 0.059, *p* = 0.055). The model explained 78% of the variance.

**Conclusion:**

Mental health nurses' perception of the trauma specialist nurse role is more strongly associated with TIC attitudes and clear professional authority than with knowledge alone, highlighting the need for formal role definition and leadership support.

**Reporting Method:**

STROBE is the relevant reporting method that has been adhered to.

## Key Points

**What is Known on the Subject:**

- Trauma‐informed care is widely recognised as important in mental health nursing.
- Mental health nurses often care for patients with complex trauma histories.
- Trauma‐informed care is difficult to implement due to limited training and unclear professional roles.

**Originality (What the Paper Adds):**

- Professional authority and responsibility are the factors most strongly associated with support for the trauma specialist nurse role among mental health nurses.
- Positive attitudes towards trauma‐informed care are also associated with greater support for the role.
- Trauma‐related knowledge alone shows only a limited association with role acceptance.

**Significance (Implications/Impact):**

- Mental health services may benefit from clearly defining the trauma specialist nurse role and its authority.
- Strong leadership support is likely to be important for successful role implementation.
- Integrating trauma‐focused training into routine staff education may strengthen trauma‐informed practice.

**Methodological Rigour:**

This study adhered to the STROBE guidelines for cross‐sectional research and applied multiple design strategies to enhance methodological robustness. Sample size was determined a priori using G*Power to ensure adequate statistical power for multivariable analyses. All measurement instruments underwent cultural adaptation and demonstrated strong psychometric properties, including expert content validation, pilot testing, and high internal consistency. Standardised data collection procedures were used to minimise bias, and clear inclusion criteria supported internal validity. Ethical approval was obtained in accordance with the Declaration of Helsinki, ensuring transparency and integrity throughout the research process.

**Relevance Statement:**

This study provides insight into mental health nursing practice by identifying the key factors associated with nurses' support for a trauma specialist nurse role. The findings indicate that clear professional authority, defined responsibility, and trauma‐informed values are more strongly associated with role acceptance than knowledge alone. These insights may offer practical guidance for nurse leaders, service managers, and policymakers seeking to strengthen TIC implementation. By clarifying organisational prerequisites for role integration, this study may contribute to safer psychiatric environments, enhanced workforce confidence, and improved trauma‐sensitive clinical care.

## 1 Introduction

The prevalence and clinical impact of psychological trauma have gained substantial recognition within contemporary mental health systems (Kleber 2019). Over the past two decades, trauma has shifted from being understood primarily as an individual clinical experience to being conceptualised as a widespread public health phenomenon with profound psychological, behavioural, and physiological consequences (Isobel and Thomas 2022; Parker and Johnson‐Lawrence 2022). Exposure to traumatic events, including interpersonal violence, neglect, combat, forced migration, and acute psychiatric crises, has been consistently associated with increased vulnerability to depression, post‐traumatic stress disorder, emotional dysregulation, and chronic health conditions (Cruz et al. 2022; McNally 2017). Large‐scale epidemiological studies, including the ACE framework, further highlight the cumulative and long‐term effects of early‐life trauma, demonstrating its strong associations with mental and physical morbidity across the lifespan (Hughes et al. 2017).

Trauma is increasingly recognised as a defining factor in psychiatric care, shaping symptom expression, treatment responses, and service utilisation patterns, although these effects have likely been present irrespective of their formal recognition in epidemiological research (Cruz et al. 2022). Mental health nurses encounter service users whose psychiatric presentations are closely linked to trauma histories, with increasing recognition of these trauma‐related factors rather than a true increase in their prevalence (Wilson et al. 2023). In response to this need, trauma‐informed care (TIC) has emerged as a leading framework aimed at recognising the pervasive effects of trauma and integrating principles of safety, empowerment, and collaboration into mental health service delivery (Dowdell and Speck 2022). However, despite increasing awareness, trauma‐informed approaches remain unevenly implemented across psychiatric settings due to significant gaps in professional training, organisational support, and role clarity (Purkey et al. 2018).

## 2 Background

### 2.1 TIC and Its Relevance to Mental Health Nursing

TIC has emerged as a leading practice framework emphasising safety, trust, collaboration, empowerment, and cultural sensitivity (Krause et al. 2018). Its implementation requires providers to integrate trauma awareness into clinical assessment, therapeutic communication, environmental design, and organisational policy (İnci et al. 2025). Nurses, who frequently serve as the primary point of therapeutic contact in psychiatric settings, play a critical role in supporting recovery, responding to trauma‐related behaviours, and ensuring that care does not contribute to re‐traumatisation (Wilson et al. 2023). However, despite its strong conceptual and ethical foundation, growing evidence indicates that the integration of TIC into routine mental health nursing practice remains inconsistent (Purkey et al. 2018). This inconsistency reflects a persistent gap between the widespread endorsement of TIC principles and their practical application in everyday clinical settings.

### 2.2 Challenges in Implementing TIC in Practice

Despite growing global endorsement of TIC, empirical studies reveal substantial barriers to its implementation. A qualitative study by Wilson et al. (2023) found that mental health nurses often endorse trauma‐informed principles but feel inadequately trained, emotionally overwhelmed, or uncertain about their clinical authority in trauma‐related interventions. This gap has been associated with the continued use of coercive measures, although the relationship is likely complex and influenced by multiple clinical and organisational factors (Heffernan et al. 2024). These findings highlight an ongoing tension in mental health nursing between trauma‐informed values and the realities of clinical practice, particularly in high‐acuity and resource‐constrained environments, where safety, risk management, and institutional demands may conflict with trauma‐sensitive approaches (Sweeney et al. 2016).

Similarly, a survey conducted among psychiatric rehabilitation nurses in Malta demonstrated that although participants expressed positive attitudes towards TIC, many struggled to define TIC, identify trauma manifestations, or perceive trauma management as part of their formal responsibilities. These findings point to structural ambiguity, insufficient training, and role uncertainty within mental health nursing (Cilia Vincenti et al. 2022). Taken together, the literature suggests that barriers to TIC implementation are not solely related to knowledge deficits, but may also involve organisational factors, professional boundaries, and unclear role expectations, as suggested in previous literature (Purkey et al. 2018; Sweeney et al. 2016).

### 2.3 The Emerging Role of the Trauma Specialist Nurse

International literature increasingly highlights the need for a formal trauma‐specialised nursing role capable of integrating neurobiological, psychosocial, and cultural trauma knowledge into multidisciplinary care (Dowdell and Speck 2022). According to Cilia Vincenti et al. (2022), trauma specialist nurses not only provide direct clinical care but also contribute to staff supervision, trauma screening, service planning, and organisational leadership in trauma‐informed initiatives. Recent discussions in the literature suggest that such specialist roles may serve as potential facilitators in supporting the implementation of trauma‐informed principles at the organisational level (Isobel and Thomas 2022; Sweeney et al. 2016).

However, the role remains underdefined in many countries. In Israel, no structured training pathway, regulatory framework, or formal job description currently exists for trauma‐specialised mental health nurses. Preliminary conceptual work suggests that such a role has been proposed as a potential approach to improving patient outcomes, reducing coercion, and enhancing staff wellbeing; however, robust empirical evidence supporting its effectiveness remains limited (Young et al. 2025). The current literature has not yet established the effectiveness of trauma specialist nurse roles through rigorous experimental designs, and further research, particularly randomised controlled trials, is required to evaluate their clinical and organisational impact. Accordingly, the introduction of trauma specialist nurse roles should be considered an exploratory development, and their implementation warrants rigorous empirical evaluation rather than being assumed to represent an established or evidence‐based solution.

Despite these conceptual advances, there is limited empirical evidence examining how mental health nurses perceive this emerging role and which factors influence their support for its implementation. This gap is particularly evident in the Israeli context, where TIC is increasingly recognised, yet formalised role structures and professional definitions remain underdeveloped.

Therefore, the aim of this study was to examine mental health nurses' perceptions of the role of the trauma specialist nurse, including their understanding of the role's professional definition, functional significance, scope of authority, and required competencies. In line with the existing literature on TIC and the importance of role clarity and professional authority in nursing practice, the following hypotheses were formulated:

1. Positive attitudes towards TIC will be positively associated with favourable perceptions of the trauma specialist nurse role.
2. Perceived professional authority and responsibility will be positively associated with favourable perceptions of the trauma specialist nurse role.
3. Trauma‐related knowledge and skills will be positively associated with favourable perceptions of the trauma specialist nurse role, although to a lesser extent than attitudes and professional authority.

## 3 Methods

### 3.1 Study Design

This cross‐sectional study examined mental health nurses' perceptions of the role, scope, and required competencies of the trauma specialist nurse. The study adhered to the STROBE guidelines for observational research. A structured, self‐administered questionnaire was used to assess demographic variables, attitudes towards TIC, perceived professional responsibilities, trauma‐related competencies, and conceptualisation of the trauma specialist nurse role.

A cross‐sectional design was selected as it allows for the efficient examination of associations between multiple variables within a relatively large sample, providing a broad overview of current perceptions and attitudes. This design was considered appropriate given the exploratory nature of the study and its aim to identify predictors rather than to establish causality or explore in‐depth individual experiences.

### 3.2 Participants and Data Collection

A statistical power analysis was conducted using G*Power Version 3.1.9.2 to determine the minimum required sample size for multiple regression analysis. With an alpha level of 0.05, a power of 0.95, and a medium effect size (*f* ^2^ = 0.15), based on Cohen's (1988) conventions, and ten predictor variables, the analysis yielded a minimum required sample of 137 participants. To accommodate a potential 10% attrition rate, the target recruitment was set at 152 nurses. Data collection was carried out between May and July 2025 through an online anonymous survey disseminated to registered mental health nurses working in inpatient psychiatric hospitals, community mental health centres, rehabilitation units, and acute psychiatric wards throughout Israel. Participants were recruited through professional nursing networks, institutional mailing lists, and direct distribution via nurse managers across multiple clinical settings, in order to ensure broad access to eligible participants.

Nurses were eligible to participate if they were registered mental health nurses, possessed at least one year of clinical experience in psychiatric settings, were able to read Hebrew, and provided voluntary informed consent. The requirement of at least one year of clinical experience was intended to ensure sufficient professional exposure to trauma‐related care and organisational practices. Nurses with less than one year of psychiatric experience or those not currently practising in mental health settings were excluded. Additional exclusion criteria included non‐nursing staff and nursing students to ensure a homogeneous professional sample.

Participation was voluntary, anonymous, and independent of workplace evaluation. To ensure anonymity, no identifying information (such as names, workplace identifiers, or IP addresses) was collected, and participation was completed through a secure online platform.

Although convenience sampling was used, several steps were taken to reduce sampling bias. Nurses were recruited from a wide range of psychiatric settings across Israel to enhance diversity and improve representativeness. To minimise social desirability bias, participants were assured of full anonymity and informed that responses would be used solely for research purposes. In addition, the use of a self‐administered online questionnaire reduced interviewer influence.

The use of an anonymous, standardised online survey further minimised social desirability bias and ensured consistency in data collection procedures. All participants completed the same structured questionnaire under identical conditions, ensuring consistency across settings. This sampling approach was considered appropriate given the exploratory nature of the study and its aim to capture a broad range of perspectives across diverse mental health care settings.

### 3.3 Measures

The study employed a structured self‐report questionnaire comprising four sections and a total of 45 items designed to assess mental health nurses' perceptions of the role, responsibilities, and competencies of the trauma specialist nurse. The instrument was adapted from the validated Attitudes Related to Trauma‐Informed Care (ARTIC) Scale, originally developed by Baker et al. (2016), which has demonstrated strong psychometric properties, with Cronbach's alpha values ranging from 0.82 to 0.93. The adaptation process involved the selection and modification of items from the original ARTIC scale, alongside the development of additional items tailored to the study aims and the Israeli psychiatric context. The final instrument included items across four domains: TIC attitudes, perceptions of the trauma specialist nurse role, professional authority and responsibility, and trauma‐related knowledge and skills. All items used in the final analysis are presented in Appendix A to enhance transparency and support replication.

TIC was assessed through participants' self‐reported attitudes and perceptions rather than direct observation of clinical practice, reflecting their general orientation towards trauma‐informed principles across different care settings. Given the variability of TIC implementation across clinical contexts, this approach was intended to capture a standardised measure of attitudes that is applicable across diverse mental health settings and patient populations.

### 3.4 Socio‐Demographic and Work‐Related Characteristics of Participants

This section included 11 items assessing participants' background characteristics, such as age, gender, and marital status, and examples of work‐related variables, such as years of clinical experience, employment setting, and prior exposure to the trauma specialist nurse role.

### 3.5 TIC Attitudes

This section included 10 items assessing participants' general attitudes towards TIC. The items examined beliefs about the importance of trauma‐informed practice, the skills required, and the anticipated benefits for patients and staff. Sample items include “Trauma‐informed care is essential in psychiatric treatment” and “mental health nurses need specialised knowledge and skills in trauma care.” Participants rated their agreement on a 5‐point Likert scale ranging from 1 = strongly disagree to 5 = strongly agree. Higher scores on this scale indicate more positive attitudes towards TIC.

### 3.6 Perception of Trauma Specialist Nurse Role

This section included 8 items assessing nurses' perceptions of the clinical, therapeutic, and organisational contributions of a trauma specialist nurse within psychiatric settings. The items addressed beliefs about the role's potential to enhance quality of care, prevent retraumatisation, support group‐based interventions, and require specialised training and interpersonal skills. Example items include: “A trauma specialist nurse can significantly improve the quality of care for patients with psychological trauma,” “The role of the trauma specialist nurse is important for preventing retraumatisation,” and “The trauma specialist nurse plays a key role in group‐based therapeutic processes.” Participants rated their agreement on a 5‐point Likert scale ranging from 1 = strongly disagree to 5 = strongly agree. Higher scores indicated more favourable perceptions of the trauma specialist nurse role.

### 3.7 Professional Authority and Responsibility

This section included 11 items assessing nurses' perceptions of the appropriate scope of authority, leadership responsibilities, and institutional involvement expected of a trauma specialist nurse. The items addressed views on clinical decision‐making authority, team supervision, participation in policy development, interdisciplinary collaboration, and the allocation of organisational resources to support the role. Representative items include: “The trauma specialist nurse should provide guidance and supervision to the clinical team.” “The trauma specialist nurse should support patients' rehabilitation processes,” and “The role requires collaboration with psychologists and psychiatrists.” Participants rated their agreement on a 5‐point Likert scale ranging from 1 = strongly disagree to 5 = strongly agree. Higher scores reflected greater support for an expanded and formalised professional role.

### 3.8 Trauma‐Related Knowledge and Skills

This final section included five items assessing nurses' self‐reported knowledge, preparedness, and confidence in identifying and managing psychological trauma. The items addressed awareness of trauma symptoms, familiarity with core principles of TIC, prior participation in professional training, and perceived confidence in treating trauma‐affected patients. Example items include “I know how to identify signs of psychological trauma in patients” and “I feel I have enough tools to treat patients with psychological trauma.” Participants rated their agreement on a 5‐point Likert scale ranging from 1 = strongly disagree to 5 = strongly agree. Higher scores indicated greater perceived trauma‐related knowledge and clinical confidence, rather than objectively assessed clinical competence.

### 3.9 Validity and Reliability

The original ARTIC Scale (Baker et al. 2016) has demonstrated strong psychometric properties, with reported internal consistency values ranging from *α* = 0.82 to 0.93. In the present study, the adapted instrument demonstrated excellent internal consistency (Cronbach's *α* = 0.93 for the total scale). The internal consistency of the subscales was also high, with Cronbach's alpha coefficients of *α* = 0.91 for trauma‐informed attitudes, *α* = 0.93 for perception of the trauma specialist nurse role, *α* = 0.95 for professional authority and responsibility, and *α* = 0.92 for trauma‐related knowledge and skills.

Content validity was established through systematic expert review. Three senior clinicians with advanced expertise in trauma‐informed mental health nursing independently assessed each item for relevance, representativeness, and conceptual accuracy. Their evaluations ensured that the adapted items appropriately reflected the constructs of trauma‐informed attitudes, professional authority, role perceptions, and trauma‐related competencies within the Israeli mental health context. Following this review, minor modifications were made to improve item clarity and contextual relevance, while maintaining the original conceptual structure of the instrument.

To further support face and construct validity, a pilot test with 20 mental health nurses was conducted to evaluate item clarity, linguistic appropriateness, and cultural sensitivity. Feedback from the pilot phase led to minor changes in wording and structure, which made the instrument fit better in its context. External validity (generalisability) was addressed by sampling mental health nurses from a range of inpatient, community, forensic, and rehabilitation settings across Israel. Although convenience sampling has inherent limitations, the inclusion of diverse psychiatric work environments enhances the applicability of the findings to the broader mental health nursing population. Together, these procedures support the adapted instrument's psychometric robustness, demonstrating strong reliability and multiple forms of validity consistent with standard scale‐adaptation methodologies in health sciences research.

### 3.10 Data Analysis

Data were analysed using IBM SPSS Statistics (Version 29.0). Descriptive statistics, including frequencies, percentages, means, and standard deviations, were used to summarise participants' demographic and professional characteristics and their responses to the questionnaire scales. Differences in perceptions of the trauma specialist nurse role according to demographic and professional characteristics (such as gender, years of experience, workplace setting, and prior exposure to the trauma specialist nurse role) were examined using independent *t*‐tests and one‐way analyses of variance. Pearson correlation coefficients were calculated to explore associations among the major study variables, including TIC attitudes, perception of the trauma specialist nurse role, professional authority and responsibility, and trauma‐related knowledge and skills. Multiple regression analysis using the enter method was conducted to identify predictors of favourable perceptions of the trauma specialist nurse role. Assumptions of normality and homoscedasticity were examined by inspecting residual plots and using the Kolmogorov‐Smirnov and Breusch‐Pagan tests. Multicollinearity diagnostics were assessed using variance inflation factors (VIF) and tolerance values. All VIF values were below 5 and tolerance values exceeded 0.20, indicating no evidence of problematic multicollinearity among the independent variables. Statistical significance was set at *p* < 0.05.

### 3.11 Ethical Considerations

The study was approved by the institutional ethics committee of the participating medical centre and it adhered to the principles of the Declaration of Helsinki. As the study involved an anonymous, self‐administered online questionnaire distributed directly to individual nurses, without access to institutional or patient‐level data, additional institutional approvals from other sites were not required. Recruitment was conducted at the individual level across multiple settings, and participation was entirely voluntary and independent of workplace affiliation.

Because data were collected via an online survey, an information page preceded the questionnaire, explaining the purpose of the study, the voluntary nature of participation, potential risks and benefits, and measures taken to ensure confidentiality and anonymity. Electronic informed consent was obtained from each participant before access to the questionnaire was granted. Participants were informed that they could discontinue the survey at any point without any consequences. All data were stored on password‐protected servers, and only aggregated results were reported.

## 4 Results

### 4.1 General and Work‐Related Characteristics

A total of 152 mental health nurses participated in the study. The mean age of the participants was 48.4 years (SD = 10.2), and the majority were women. Most nurses were married, had children, and were Jewish. With regard to religiosity, most identified as secular or traditional. The inclusion of religious affiliation was considered relevant given its potential influence on attitudes towards trauma, coping mechanisms, and perceptions of care, particularly within the sociocultural context of mental health services in Israel.

Participants worked across a wide range of psychiatric settings, with the largest proportion employed in closed or open inpatient wards and forensic or rehabilitation settings. Clinical settings included inpatient units (closed and open wards), acute psychiatric intake (emergency services), long‐term (chronic) care units, day hospitalisation frameworks, and community‐based mental health and rehabilitation services. On average, the nurses reported 18.6 years (SD = 12.8) of experience in the nursing profession and 8.1 years (SD = 8.0) of tenure in their current unit. Approximately three‐quarters of the sample had completed a post‐basic course in mental health nursing, and 16.4% had previously been exposed to the trauma specialist nurse role (Table 1). In the Israeli context, post‐basic mental health courses refer to advanced professional training programmes in mental health nursing that may include components related to TIC, although they are not exclusively focused on trauma.

**TABLE 1:** Socio‐demographic and work‐related characteristics of participants (*N* = 152).

Characteristic	Category/Statistic	*n*	%	*M*	SD
Age (years)	—	—	—	48.4	10.2
Gender	Men	38	25.0	—	—
	Women	114	75.0	—	—
Marital status	Single	14	9.2	—	—
	Married	110	72.4	—	—
	Divorced	28	18.4	—	—
Number of children	—	—	—	2.4	1.4
Ethnic sector	Jewish	131	86.2	—	—
	Arab	21	13.8	—	—
Religiosity	Secular	91	59.9	—	—
	Traditional	46	30.3	—	—
	Religious	13	8.6	—	—
	Ultra‐Orthodox	2	1.3	—	—
Clinical setting (department type)	Closed Ward	56	36.8	—	—
	Open Ward	36	23.7	—	—
	Emergency Department	2	1.3	—	—
	Long‐Term (Chronic) Ward	7	4.6	—	—
	Day Hospitalisation	8	5.3	—	—
	Other	43	28.3	—	—
Years in nursing	—	—	—	18.6	12.8
Years in current unit	—	—	—	8.1	8.0
Post‐basic mental health course	No	38	25.0	—	—
	Yes	114	75.0	—	—
Prior exposure to the trauma specialist nurse role	No	127	83.6	—	—
	Yes	25	16.4	—	—

Abbreviations: *M* = mean; SD = standard deviation.

### 4.2 Descriptive Statistics of the Main Study Variables

Participants reported highly positive TIC attitudes, with a mean score of 4.62 (SD = 0.40) on a 1–5 scale, indicating generally strong and positive TIC attitudes. Perception of the trauma specialist nurse role was similarly favourable (*M* = 4.52, SD = 0.57), reflecting participants' supportive views of the proposed role and its relevance to psychiatric practice. The domain of professional authority and responsibility received the highest ratings overall (*M* = 4.58, SD = 0.55), suggesting strong endorsements for a more formalised and expanded scope of authority for the trauma specialist nurse. In contrast, trauma‐related knowledge and skills demonstrated more moderate levels (*M* = 3.67, SD = 0.76), indicating relatively lower perceived confidence in trauma‐related clinical competencies (Table 2).

**TABLE 2:** Means and standard deviations of trauma‐related scales (*N* = 152).

Scale	Items (*n*)	Possible range	*M*	SD
Trauma‐informed care attitudes	10	1–5	4.62	0.40
Perception of trauma specialist nurse role	8	1–5	4.52	0.57
Professional authority and responsibility	10	1–5	4.58	0.55
Trauma‐related knowledge and skills	5	1–5	3.67	0.76

Abbreviations: *M* = mean; SD = standard deviation.

### 4.3 Differences in Perceptions According to Professional Characteristics

Analyses of group differences showed that nurses who completed a post‐basic mental health course reported significantly more favourable perceptions of the trauma specialist nurse role, with mean scores of 4.70 and 4.46 for the trained and non‐trained groups, respectively (*t*(152) = 2.72, *p* = 0.008). In contrast, prior exposure to the trauma specialist nurse role did not yield meaningful differences (*p* > 0.05).

### 4.4 Correlations Between the Main Study Variables

Pearson correlation analysis revealed strong and statistically significant associations between the main study variables. TIC attitudes were strongly correlated with perceptions of the trauma specialist nurse role (*r* = 0.77, *p* < 0.01) and with professional authority and responsibility (*r* = 0.73, *p* < 0.01). Role perceptions also correlated strongly with professional authority and responsibility (*r* = 0.86, *p* < 0.01). Correlations between trauma‐related knowledge and skills and the other domains were smaller but still positive. Specifically, trauma‐related knowledge and skills showed weak but significant correlations with perceptions of the trauma specialist nurse role (*r* = 0.24, *p* < 0.01) and with professional authority and responsibility (*r* = 0.21, *p* = 0.01). No statistically significant associations were found between age or years of professional experience and attitudes towards TIC. Similarly, no statistically significant differences were found between participants who had completed post‐basic mental health training and those who had not in relation to trauma‐related knowledge and skills (*p* > 0.05).

### 4.5 Predictors of Favourable Perception of the Trauma Specialist Nurse Role

A multiple regression analysis was conducted to identify factors that significantly predicted favourable perception of the trauma specialist nurse role (Table 3). TIC attitudes (*β* = 0.423, *p* < 0.001) and professional authority and responsibility (e.g., greater support for an expanded and formalised professional role) (*β* = 0.643, *p* < 0.001) emerged as the strongest predictors. Trauma‐related knowledge and skills demonstrated a marginal association (*β* = 0.059, *p* = 0.055). Years of experience, post‐basic training status, and prior exposure to the role did not significantly predict favourable perceptions of the trauma specialist nurse role in the multivariate model. The full regression model explained 78% of the variance in perceptions of the trauma specialist nurse role (Adjusted *R* ^2^ = 0.78).

**TABLE 3:** Multiple regression predicting perception of the trauma specialist nurse role (*N* = 152).

Predictor	*B*	SE *B*	*t*	*p*
Trauma‐informed care attitudes	0.423	0.082	5.16	< 0.001
Professional authority and responsibility	0.643	0.059	10.87	< 0.001
Trauma‐related knowledge and skills	0.059	0.030	1.94	0.055
Years in nursing	−0.000	0.000	−0.44	0.660
Post‐basic course	−0.082	0.053	−1.54	0.126
Prior exposure to the role	−0.057	0.061	−0.94	0.351

*Note: B* = unstandardised regression coefficient; SE *B* = standard error of *B*; *t* = *t*‐statistic; *p* = significance level. Model summary: *R* ^2^ = 0.79, Adjusted *R* ^2^ = 0.78, *F*(6, 142) = 87.53, *p* < 0.001.

## 5 Discussion

The findings of this study explored how mental health nurses perceive the proposed trauma specialist nurse role, including their views on its professional definition, scope of authority, and required competencies. These aspects were assessed through structured questionnaire items addressing role perceptions, professional authority and responsibility, and trauma‐related knowledge and skills, as detailed in the Measures section. Overall, participants expressed positive TIC attitudes and viewed a trauma specialist nurse as potentially beneficial in psychiatric settings.

The present study does not provide evidence regarding the effectiveness of the trauma specialist nurse role. Rather, it identifies factors associated with nurses' support for this emerging role. Future research, particularly using intervention‐based and experimental designs such as randomised controlled trials, is required to evaluate the clinical and organisational impact of a clearly defined trauma specialist nurse role.

Consistent with the study hypotheses, the regression analysis demonstrated that professional authority and responsibility, followed by TIC attitudes, were the strongest predictors of favourable perceptions of the trauma specialist nurse role, whereas trauma‐related knowledge and skills showed only a marginal association. These findings indicate that nurses' support for a dedicated trauma specialist role is more closely associated with their understanding of the role's formal scope and authority, as well as their orientation towards trauma‐informed principles, than with their self‐assessed knowledge or training in trauma care. In this study, such values and beliefs were reflected in the TIC attitudes scale.

A key interpretation emerging from these findings is that mental health nurses appear to prioritise structural and organisational enablers, particularly clear professional authority and responsibility, over individual capabilities when evaluating new professional roles. This tendency is consistent with broader literature indicating that specialist nursing roles gain legitimacy primarily when they are embedded within clearly defined professional hierarchies, supported by organisational policy, and accompanied by visible leadership support (Torrens et al. 2020). In this sense, the trauma specialist nurse is perceived not only as a clinical expert but as an institutional anchor capable of shaping trauma‐informed culture.

Importantly, nurses who held more favourable TIC attitudes were significantly more likely to view the trauma specialist nurse role in a positive light. This aligns with the idea that embracing trauma‐informed principles creates readiness to support specialised roles that promote those principles. Those who recognise the profound impact of trauma on mental health and the need for sensitive, holistic care tend to welcome an advanced practice role focused on trauma. Interestingly, no significant associations were found between age or years of professional experience and attitudes towards TIC. These findings suggest that attitudes towards TIC are not strongly associated with demographic or experience‐related variables and may be more closely aligned with broader professional values and orientations towards trauma‐informed practice (Purkey et al. 2018; Sweeney et al. 2016).

Moreover, the perception that a trauma specialist nurse should have clear authority and defined responsibilities within the clinical team was also a key factor in accepting the role. In fact, this was the strongest predictor in the regression model. In this study, professional authority and responsibility refer to nurses' perceptions of the scope of decision‐making authority, leadership roles, and involvement in clinical and organisational processes attributed to the trauma specialist nurse role. In other words, participants who believed that this specialist nurse would be empowered, for example, to guide staff training, conduct trauma‐sensitive assessments, or lead programme development, were more inclined to endorse the role's importance. This finding highlights the central significance of professional authority and responsibility. Namely, mental health nurses appear to value the trauma specialist position most when it is envisioned as a well‐defined, authoritative role rather than an ambiguous add‐on duty. It echoes prior qualitative findings that lack of formal recognition and clear scope can hinder nurses' willingness to adopt trauma‐focused practices (Wilson et al. 2023). In settings where roles and responsibilities for TIC are unclear, nurses may feel it is “not their place” to take initiative, underscoring the need for organisational frameworks that legitimise and support trauma specialists.

This emphasis on authority also reflects long‐standing challenges in mental health nursing, where role confusion and limited decision‐making autonomy have repeatedly been identified as barriers to implementing evidence‐based practices (Byrt et al. 2018). A clearly defined trauma specialist role may therefore address not only clinical needs but also professional identity needs within the mental health nursing workforce.

By contrast, trauma‐related knowledge and skills did not substantially influence how nurses perceived the specialist role. This finding may reflect relatively homogeneous or limited exposure to trauma‐specific education within the sample, consistent with the absence of standardised trauma training in Israeli nursing curricula and routine in‐service education. This interpretation is further supported by the absence of differences between nurses with and without post‐basic mental health training in relation to trauma‐related knowledge and skills, suggesting that formal training alone may not be sufficient to meaningfully differentiate perceived competence in trauma care. Internationally, it has been reported that many mental health professionals lack familiarity with TIC concepts and feel insufficiently prepared to deliver trauma‐specific interventions (İnci et al. 2025).

Another interpretation is conceptual, suggesting that knowledge may influence individual preparedness but may not shape attitudes towards system‐level roles. This is consistent with literature suggesting that professional role perceptions are more strongly shaped by organisational culture, structural factors, and perceived legitimacy than by individual knowledge alone (Duyilemi and Mabunda 2025; Purkey et al. 2018; Sweeney et al. 2016). Thus, knowledge alone may be insufficient to influence perceptions of a new specialist role unless coupled with strong organisational support and cultural endorsement. This finding should be interpreted with caution, as trauma‐related knowledge and skills were assessed based on self‐reported perceptions rather than objective measures of clinical competence.

When comparing these findings to previous research, several parallels and contrasts emerge. Participants' positive TIC attitudes align with findings from other psychiatric settings. For example, a survey of mental health nurses in Malta found broadly favourable views of trauma‐informed approaches (Cilia Vincenti et al. 2022). However, that same body of work highlighted an important gap. Namely, many nurses did not feel authorised or responsible for implementing trauma‐focused interventions in practice (Cilia Vincenti et al. 2022; Nikopaschos et al. 2025). This is consistent with our finding that professional authority and responsibility were the strongest statistical predictors of nurses' perception of the trauma specialist nurse role. Without a clear mandate or designated role, mental health nurses may hesitate to take on trauma‐focused initiatives even when their TIC attitudes are positive. In the Maltese context, nurses who lacked a defined structure for TIC tended to default to custodial or control‐based approaches rather than trauma‐sensitive engagement (Cilia Vincenti et al. 2022; Nikopaschos et al. 2025). Our results reinforce the idea that establishing a recognised trauma specialist nurse role may help address this gap by potentially providing a clear leader and resource person to guide trauma‐informed practice, potentially reducing reliance on coercive interventions. This interpretation is supported by Wilson et al. (2023), who found that mental health nurses in acute care environments often felt insecure about applying TIC due to limited support and training, at times resorting to practices such as seclusion or restraint despite concerns about re‐traumatisation. In our study, nurses with stronger TIC attitudes (and presumably greater openness to trauma‐informed approaches) were more supportive of the specialist role, but they placed greatest weight on formal authority and responsibility, suggesting that enhanced training, role clarity, and leadership may be essential to transforming practice. Prior interventions have shown that structured trauma education and visible trauma‐informed leadership can improve therapeutic engagement and reduce coercive practices in inpatient settings (Heffernan et al. 2024). Thus, our findings contribute to growing evidence that while frontline mental health nurses understand the importance of TIC, effective implementation requires the right infrastructure, designated roles, and sustained organisational support.

The strong correlations observed between TIC attitudes, professional authority and responsibility, and perceptions of the trauma specialist nurse role may reflect conceptual proximity between these constructs, as they all relate to a broader professional orientation towards trauma‐informed practice. Particularly, the high correlations observed (*r* > 0.80) may indicate shared variance between constructs, suggesting the need for further dimensional refinement in future research. At the same time, the use of theoretically grounded and separately operationalised subscales supports their conceptual distinction. Nevertheless, the possibility of partial construct overlap cannot be fully excluded, particularly given the reliance on self‐reported measures. Future studies employing factor analytic approaches are recommended to further examine construct distinctiveness and refine measurement precision.

Finally, this study is among the first to investigate nurses' perception of the trauma specialist nurse role in Israel, where such a position is not yet formally established. The positive reception of this role, combined with the dominant influence of professional authority and responsibility and the strong contribution of TIC attitudes, provides important guidance for nursing leadership. It suggests that mental health nurses are open to innovation, yet successful adoption of a new trauma specialist role will require attention to both attitudinal and especially structural factors. Simply increasing trauma‐related knowledge and skills through isolated trainings is unlikely to be sufficient. Instead, building a trauma‐informed organisational culture, providing ongoing education, and clearly delineating the trauma specialist nurse's authority and responsibilities will be essential. These findings suggest that integrating trauma‐focused training into routine staff education may strengthen trauma‐informed practice and should be considered in future educational and organisational interventions. Our findings therefore highlight the importance of both individual‐level preparation (education, confidence building, and attitude development) and organisational‐level changes (role definition, policy integration, and leadership support) in advancing trauma‐informed mental health nursing practice.

### 5.1 Limitations

This study has several limitations that should be considered when interpreting the findings. First, the study relied on self‐reported perceptions, which may not fully reflect actual trauma‐related competencies or clinical behaviours and may be influenced by social desirability bias. Although the use of a structured and theory‐informed questionnaire enhanced measurement consistency, the reliance on self‐report remains an important consideration when interpreting the findings. Moreover, the cross‐sectional design precludes causal interpretation of the observed associations between the study variables. In addition, the use of convenience sampling through online recruitment may have limited the representativeness of the sample and introduced selection bias, particularly favouring nurses with greater interest in trauma‐informed care.

While participants were recruited from a range of inpatient and community mental health settings, representation of private sector services was limited, likely due to the recruitment methods based on voluntary participation through professional networks and institutional distribution. Consequently, the limited inclusion of community‐based and private psychiatric settings may restrict the generalisability of the findings.

Furthermore, although the adapted questionnaire demonstrated good internal consistency, trauma‐related knowledge and skills were assessed using a small number of self‐reported items reflecting perceived competence rather than objective clinical performance. This may have reduced measurement precision and contributed to the relatively modest predictive value observed for this variable.

The requirement for Hebrew language proficiency may have introduced selection bias, particularly with regard to the underrepresentation of non‐Hebrew‐speaking populations, including some Arab Israeli nurses. However, Hebrew is the primary language used in mental health services and professional communication in Israel, which may reduce the extent of this limitation.

Finally, because the trauma specialist nurse role is not yet formally defined in Israel, participants may have interpreted the role differently. In light of these limitations, future research using longitudinal designs, validated behavioural measures, and broader sampling across diverse psychiatric settings is warranted.

### 5.2 Implications

The findings suggest that support for the implementation of a trauma specialist nurse role is more strongly associated with clear professional authority and organisational responsibility than with knowledge alone. Mental health services may therefore consider prioritising formal role definition, including decision‐making authority, leadership functions, and team supervision responsibilities. Embedding this role within institutional policy frameworks may support staff engagement with trauma‐informed practice and contribute to organisational accountability.

The relatively modest contribution of trauma‐related knowledge and skills highlights the potential importance of integrating trauma‐focused content into undergraduate and post‐basic mental health nursing education. Given that professional authority and responsibility, rather than knowledge alone, were most strongly associated with support for the trauma specialist nurse role, educational programmes may benefit from combining theoretical foundations of trauma with applied components, including leadership, ethical decision‐making, and interprofessional collaboration.

Ongoing in‐service education may reinforce both trauma‐informed values and awareness of the potential scope of the specialist role. These recommendations should be interpreted as practice and policy implications derived from the present findings, rather than as evidence of causal effects, given the cross‐sectional design of the study.

Future studies should examine the longitudinal impact of implementing a formal trauma specialist nurse role on staff wellbeing, use of coercive interventions, patient outcomes, and organisational culture. Mixed‐methods and intervention‐based designs are recommended to evaluate how professional authority, policy integration, and leadership endorsement are associated with real‐world trauma‐informed practice. In particular, future intervention studies are needed to evaluate the effectiveness of such educational and organisational strategies.

## 6 Conclusion

This study demonstrates that professional authority and responsibility, together with TIC attitudes, are the factors most strongly associated with mental health nurses' support for a trauma specialist nurse role. These findings suggest the importance of structured role definition and organisational support in advancing trauma‐informed mental health nursing practice.

## 7 Relevance to Practice

The findings of this study may inform clinical practice by suggesting that the implementation of trauma‐informed care is unlikely to rely solely on individual knowledge or goodwill, but may also require formal leadership structures and clearly defined professional authority. These insights are derived from the Israeli mental health context, where a formal trauma specialist nurse role is not yet established and therefore highlight considerations for structured role development within this system.

Mental health organisations may consider incorporating the trauma specialist nurse role into clinical governance frameworks, safety protocols, and multidisciplinary treatment pathways. Clear role designation may support staff confidence, reduce role ambiguity, enhance trauma‐sensitive decision‐making, and contribute to safer work environments and workforce protection in psychiatric settings.

## Funding

The authors have nothing to report.

## Disclosure

*Authorship statement*: All authors meet the ICMJE authorship criteria: (1) substantial contribution to study conception, design, data acquisition, or analysis; (2) drafting or critically revising the manuscript; (3) final approval of the version to be published; and (4) agreement to be accountable for the accuracy and integrity of all aspects of the work.

## Ethics Statement

The study was approved by the Helsinki Committee of Hillel Yaffe Medical Center (Approval No. 0138‐25HYMC). All participants provided informed, electronic consent prior to participation.

## Consent

The authors have nothing to report.

## Conflicts of Interest

The authors declare no conflicts of interest.

## Data Availability

The data that support the findings of this study are available from the corresponding author upon reasonable request.

## Appendix A Adapted Questionnaire Based on the ARTIC Scale (Baker et al. 2016)

The following questionnaire was used to assess mental health nurses’ perceptions of the trauma specialist nurse role. The instrument was adapted from the Attitudes Related to Trauma‐Informed Care (ARTIC) Scale (Baker et al. 2016) and culturally tailored to the Israeli psychiatric context.

### Section 1: Socio‐Demographic and Professional Characteristics

Participants were asked to report the following:

1. Age (years).
2. Gender (male/female).
3. Marital status (single/married/divorced/separated).
4. Number of children.
5. Sector (Jewish/Arab).
6. Religious affiliation (secular/traditional/religious/ultra‐Orthodox).
7. Current work setting (closed ward/open ward/emergency/long‐term care/day hospital/other).
8. Years of experience in nursing.
9. Years of experience in the current unit.
10. Completion of post‐basic mental health training (yes/no).
11. Prior exposure to the trauma specialist nurse role (yes/no).

### Response Format for Sections 2–5

Participants rated their agreement with each statement using a 5‐point Likert scale:

1 = strongly disagree, 2 = disagree, 3 = neutral, 4 = agree, 5 = strongly agree.

#### Section 2: Attitudes Towards Trauma‐Informed Care

12 Trauma‐informed care is essential in psychiatric treatment.
13 Psychiatric nurses require specialised knowledge and skills in trauma care.
14 Trauma‐informed care prevents deterioration in patients’ condition.
15 Trauma‐informed care reduces staff frustration.
16 It is important that all mental health staff understand and support trauma‐informed care.
17 I support the implementation of a trauma specialist nurse role in psychiatric wards.
18 There is a need for greater awareness of psychological trauma among mental health staff.
19 Trauma‐informed care should also involve the patient's family and environment.
20 Trauma care is a long‐term process requiring advanced skills.
21 Psychiatric nurses should consider trauma care as part of their professional role.

#### Section 3: Perceptions of the Trauma Specialist Nurse Role

22 A trauma specialist nurse can significantly improve the quality of care for patients with psychological trauma.
23 The role is important in preventing re‐traumatisation.
24 The role requires specialised training and certification.
25 The trauma specialist nurse can improve group‐based care processes.
26 Clear criteria should define who is qualified for this role.
27 Strong interpersonal and communication skills are essential for this role.
28 The role includes working with patients with histories of sexual abuse.
29 The trauma specialist nurse plays a key role in integrated treatment approaches.

#### Section 4: Professional Authority and Responsibilities

30 The trauma specialist nurse should provide staff training and supervision.
31 The role should include clinical decision‐making authority.
32 The role should be involved in institutional policy development.
33 The trauma specialist nurse should support patients’ rehabilitation processes.
34 The role requires collaboration with psychologists and psychiatrists.
35 Dedicated time or compensation should be allocated for this role.
36 Formal regulation of the role is needed.
37 The role includes supporting clinical staff.
38 The trauma specialist nurse should be involved in patient intake assessments.
39 The role includes leading rehabilitation initiatives.
40 Resources should be allocated for professional development.

#### Section 5: Trauma‐Related Knowledge and Skills (Perceived Competence)

41 I know how to identify signs of psychological trauma in patients.
42 I have sufficient tools to treat patients with psychological trauma.
43 I am familiar with the principles of trauma‐informed care.
44 I have participated in professional training in trauma care.
45 I feel confident treating patients with trauma.
